# A new CARD9 mutation (R101S) in a Brazilian patient with DEEP dermatophytosis

**DOI:** 10.1186/1939-4551-8-S1-A77

**Published:** 2015-04-08

**Authors:** Anete Grumach

**Affiliations:** 1 Médica Especialista Em Alergia e Imunologia, Faculty of Medicine ABC, Brazil

## Background

Deep dermatophytosis had been described in HIV and immunosupressed patients. Recently, the association with autosomal recessive CARD9 deficiency was found in individuals previously classified as “immunocompetent”. We describe a new CARD9 mutation associated with dermatophytosis.

## Methods

We report a 24-year-old Brazilian male with deep dermatophytosis with *Trichophyton mentagrophytes* isolated from the skin lesions. Opsonophagocyosis of Candida was performed. *CARD9*was amplified with specific primers.

## Results

The symptoms initiated with oral candidiasis at 3 years old, generalized afterwars and treated with oral and local therapy. At 11 years old well delimitated, descamative and pruriginosus skin lesions appeared; ketoconazol and itraconazole were maintained for 5 years. At 14 years old, the lesions were ulcerative, secretive and painful in the shoulders (15cm of diameter); terbinafine and posaconazole were used without result. His brother presents superficial dermatophytosis. A homozygous mutation in *CARD9 exon 3*(R101S) was identified in the patient. His parents, one brother (with superficial dermatophytosis) and one sister are heterozygous for this mutation. Laboratory evaluations showed eosinophilia and high IgE levels; Candida killing was clearly impaired in the patient.

## Conclusions

This is the first report of CARD9 deficiency in a Brazilian family and the first report of a CARD9 R101S mutation. A different mutation affecting the same amino-acid (R101C) had been previously described in two Moroccan siblings with deep dermatophytosis.

